# The central fibre areas in the tibial footprint of the posterior cruciate ligament show the highest contribution to restriction of a posterior drawer force—A biomechanical robotic investigation

**DOI:** 10.1002/jeo2.70174

**Published:** 2025-02-17

**Authors:** Adrian Deichsel, Thorben Briese, Wenke Liu, Michael J. Raschke, Alina Albert, Christian Peez, Elmar Herbst, Christoph Kittl

**Affiliations:** ^1^ Department of Trauma, Hand and Reconstructive Surgery University Hospital Münster Münster Germany

**Keywords:** biomechanics, fibre areas, posterior cruciate ligament, reconstruction

## Abstract

**Purpose:**

The purpose of this study was to determine the role of different fibre areas of the tibial footprint of the posterior cruciate ligament (PCL) in restraining posterior tibial translation.

**Methods:**

A sequential cutting study on cadaveric knee specimens (*n* = 8) was performed, utilizing a six‐degrees‐of‐freedom robotic test setup. The tibial attachment of the PCL was divided into nine areas, which were sequentially cut in a randomized sequence. After determining the native knee kinematics with 89 N anterior, and posterior tibial translation force at 0°, 30°, 60° and 90° knee flexion, a displacement‐controlled protocol was performed replaying the native motion. Utilizing the principle of superposition, the reduction of the restraining force represents the contribution (in‐situ forces) of each cut fibre area.

**Results:**

The PCL was found to contribute 25.3 ± 11.1% in 0° of flexion, 49.7 ± 19.2% in 30° of flexion, 58.9 ± 19.3% in 60° of flexion and 50.6 ± 15.1% in 90° of flexion, to the restriction of a posterior drawer force. Depending on the flexion angle, every cut area of the tibial PCL footprint was shown to be a significant restrictor of posterior tibial translation (*p* ≤ 0.05). When investigating the fibre areas from anterior to posterior, the central fibre areas showed the highest contribution (35.0%–44.3%). When investigating the fibre areas from medial to lateral, the lateral fibre areas showed the highest contribution (41.4%–43.6%) from 0 to 30° knee flexion, while the medial fibre areas showed the highest contribution (41.5%) in 90° knee flexion.

**Conclusion:**

The central row areas in the tibial footprint of the PCL were identified to be the main contributors inside the tibial footprint, while, depending on the flexion angle, the medial or lateral column fibre areas showed a higher contribution. These findings might inform the clinician to place a PCL graft centrally into the tibial footprint during reconstruction.

**Level of Evidence:**

Not applicable.

**Abbreviations**
ACL: anterior cruciate ligament
ALB: anterolateral bundle
CI: confidence interval
MD: mean difference
PCL: posterior cruciate ligament
PCLR: posterior cruciate ligament reconstruction
PMB: posteromedial bundle

## INTRODUCTION

The posterior cruciate ligament (PCL) is mostly described as a double‐bundle structure [2, 15]. However, different structural variants are described, ranging from multiple bundles to continuous, ribbon‐like ligaments [9, 25, 35, 40]. From biomechanical data, it is generally accepted that the PCL is the major restraint to posterior tibial translation. However, both bundles were found to be relevant stabilizers of the knee, and insufficiency of both bundles has to be present for a relevant posterior instability [1, 23, 27, 43]. This indicates that different fibre areas across the PCL are important for restraining a posterior drawer.

To restore the native function of the PCL‐deficient knee as closely as possible, a PCL reconstruction (PCLR) mimicking the native restraint would be favourable [43, 44]. However, the optimal tibial tunnel position for PCLR is still controversially debated [4, 8, 42, 50, 52, 53]. Different reconstruction techniques are described for the tibial side, ranging from single‐bundle to double‐bundle reconstructions, as well as tibial inlay techniques [8, 34, 44, 49, 52]. For the anterior cruciate ligament (ACL), specific fibre areas of the femoral and tibial footprint were biomechanically shown to be the primary contributors in restraining an anterior tibial translation force, advising the clinician on where to best position the tibial tunnel [26, 33, 47]. Analogous, different fibre areas in the tibial footprint of the PCL may possibly show different contributions to restrain posterior tibial translation. However, whether this is the case is currently unknown. The purpose of this study was to investigate different fibre areas of the tibial footprint of the PCL regarding their contribution to resisting a posterior tibial translation force. It was hypothesized, corresponding to the biomechanical study of the ACL [33], that fibre areas with higher contribution can be identified in the tibial footprint of the PCL, which act as the main stabilizers, against posterior tibial translation.

## MATERIAL AND METHODS

Eight unpaired cadaveric knee specimens (mean age 66.8 ± 7.6 years, range 58–77 years, three female, five male, four left, four right) without prior knee surgery, high‐grade osteoarthritis or meniscal injury, were obtained from MedCure (Portland). The experiments were performed with permission from the institutional review board of the University of Münster (IRB reference number 2023‐407‐f‐S). Before each test, the knee was controlled for injuries not specified in the testing protocol, and discarded, if necessary.

Specimens were stored at –20°C and thawed for 24 h at room temperature, before preparation. The skin and subcutaneous fat were resected, leaving the rest of the soft tissues intact. Tibia and femur were secured in aluminium cylinders, 12 cm above and below the joint line, with three‐component polyurethane bone cement (RenCast®; Gößl & Pfaff). The fibula was then cut 10 cm below the joint line and transfixed with a 3.5 mm cortical screw to the tibia [45]. Specimens were wrapped in moist tissue papers to prevent drying [54].

### Robotic test setup

A validated test setup consisting of six‐degrees‐of‐freedom industrial robot (KR 60‐3; KUKA Robotics) equipped with a force‐torque sensor (FTI Theta; ATI Industrial Automation) was used for biomechanical testing in this study, as previously described [11, 12, 13, 14]. The robotic system allows for position‐controlled movement with an accuracy of ±0.06 mm, as well as force‐controlled movement with an accuracy of ±0.25 N and ±0.05 Nm, respectively. The test setup was driven by the custom software simVITRO (Cleveland Clinic BioRobotics Lab), which optimizes the robot test setup for the simulation and acquisition of knee joint kinematics. A tactile measuring arm (Absolute Arm 8320‐7; Hexagon Metrology GmbH) with an accuracy of 0.05 mm was utilized to define landmarks on the femur and tibia plateau and shaft from which a modified Grood and Suntay coordinate system was defined [22, 41]. Data acquisition was performed with a sampling rate of 500 Hz.

### Biomechanical testing

To obtain tissue hysteresis, each specimen was flexed and extended 10 times [39]. The starting point of each knee was determined by manually minimizing all forces and torques acting on the knee in full extension. The passive path of the knee was then determined by flexing each knee from full extension to 90° of flexion while minimizing forces and torques in all axes aside from the flexion‐extension axis. During the determination of the passive path, an axial compression force of 50 N was applied to keep contact between the femur and tibia. A force‐controlled testing protocol (recording displacements in response to given forces/torques), applying 89 N anterior tibial translation force and 89 N posterior tibial translation force, replicating the forces applied by the KT1000 arthrometer [10], was performed in 0°, 30°, 60° and 90° of flexion under axial compression of 200 N. The motion of the native knee was then transferred to a position‐controlled test protocol (recording forces in response to given displacements) [54].

Utilizing the principle of superposition, the reduction of force, after replaying the native knee motions, indicated the contribution (in‐situ forces) of each cut in restricting anterior/posterior tibial translation [6, 19, 46].

### Sequential cutting protocol

**Figure 1:**
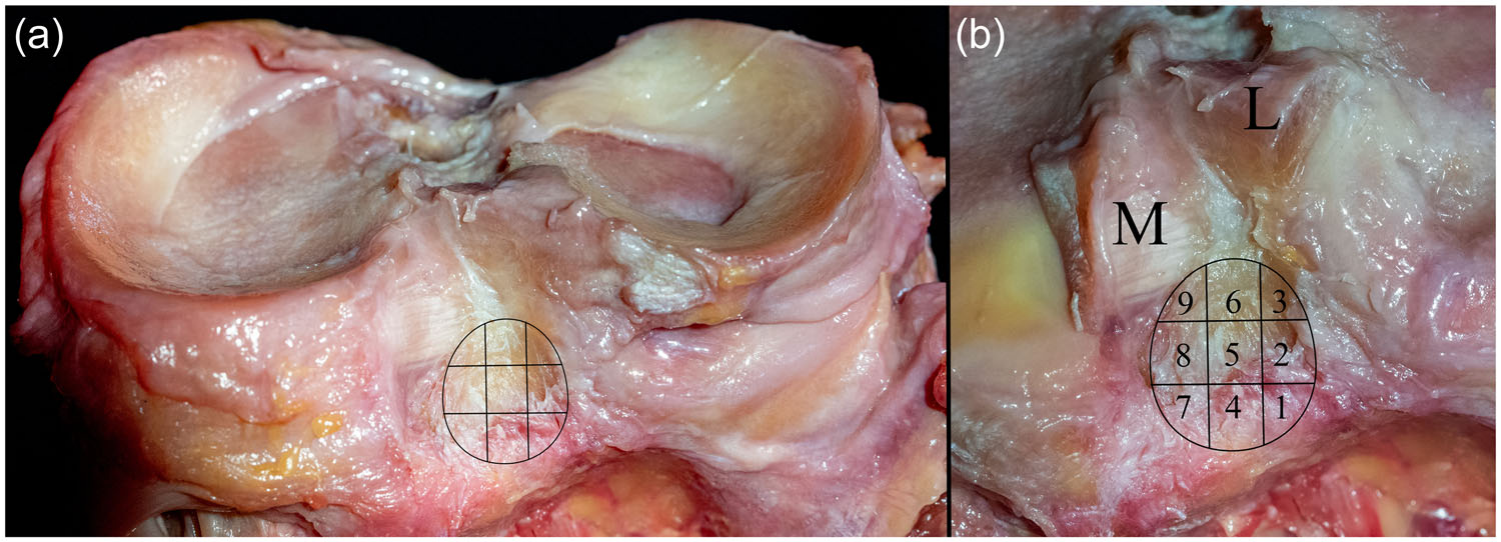
Right tibia from posterior. (a) Exemplary grid depicting sequential cutting steps of the tibial footprint of the posterior cruciate ligament. (b) The tibial footprint was divided into nine areas arranged in three columns, and three rows. Cutting was performed from posterior to anterior, starting either at the most lateral (1–3), or most medial row (7–9). L, posterior root of the lateral meniscus; M, posterior root of the medial meniscus.

A posterior approach to the tibial footprint of the PCL was established by longitudinally splitting the posterior joint capsule at the centre of the tibial width, between the oblique popliteal ligament and the superior border of the popliteus muscle. If present, the posterior meniscofemoral ligament, connecting the posterior horn of the lateral meniscus to the femoral footprint of the PCL, was cut, to exclude its influence on the sequential cutting of the tibial footprint. After the establishment of the approach, the native knee kinematics were determined, using the position‐controlled test protocol. Before starting sequential cutting, the length and width of the tibial footprint were measured using the tactile measuring arm. Based on the measurements, a grid was calculated dividing the tibial footprint into nine areas (Figure 1). Depending on the size and shape of the tibial footprint of the PCL, the grid was adjusted in shape for every specimen. In each subsequent cutting step, one single area was cut to determine its contribution to resisting knee motion, resulting in nine total cutting steps. Cutting was performed starting either with the most lateral (number 1, followed by number 2 and number 3), or most medial (number 7, followed by number 8 and number 9) areas in a randomized fashion, to minimize the effect of a potential load sharing effect across the footprint [26, 33].

### Quantification of the tibial footprint

After biomechanical testing, each tested knee was ex articulated and the peripheral soft tissues at the femur were completely resected. The complete surface of the tibial footprint of the PCL was digitized using the tactile measuring arm. Calculation of the dimensions and surfaces was performed using the PC‐DMIS software (Hexagon Metrology GmbH).

### Data Analysis, statistics and sample size calculation

Extraction of knee kinematics from the raw data of SimVitro was performed using Matlab (version R2020a, MathWorks) and Excel (Microsoft). Statistical analysis was performed using PRISM (version 10, GraphPad Software). The contribution of the complete PCL to restraining posterior tibial translation is presented as a percentage of the total forces determined in the native state (89 N). The contribution of each single area of the tibial PCL footprint is presented as a percentage of the contribution of the total PCL. The loss of force during the application of an ATT force served as an internal control. The comparison of the total contribution of the PCL in different flexion angles was performed utilizing a repeated‐measures one‐way ANOVA. Subsequently, mixed linear models were used to assess the main effects and interactions of each independent variable (cutting state in different flexion angles), with the dependent variable being the posterior tibial translation. Post hoc pairwise comparisons were used to compare the contribution of each cutting state against the intact PCL state (0% loss of force). Group means are presented as ±standard deviations (SD). Between‐group differences are presented as mean differences (MD) with corresponding 95% confidence intervals (CI). Post hoc Dunn's correction was performed to account for multiple testing. A *p* < 0.05 was deemed to identify significant differences.

To further investigate possible important clusters inside the tibial footprint, the cut areas were first grouped into three rows (posterior (areas 1/4/7), central (areas 2/5/8) and anterior (areas 3/6/9)). Furthermore, grouping into columns (lateral (areas 1/2/3), central (areas 4/5/6) and medial (areas 7/8/9) was performed.

To investigate the effect of the cutting sequence, the influence of the cutting sequence was assessed using mixed linear models with the flexion angle and the type of cutting sequence (starting either from lateral or medial) as the independent variables.

To calculate the sample size necessary for the present study, an a‐priori power analysis was performed using G*Power (version 3.1) [16]. Based on previous studies investigating the influence of different fibre bundles on the in‐situ forces of cruciate ligaments [26, 33], a sample size of *n* = 8 was calculated to show a 10% contribution of a cutting step (assuming an SD of 10%; effect size = 1), with a power of 80%, at the significance level of *p* < 0.05.

## RESULTS

No specimen had to be excluded, resulting in every tested knee being available for final analysis. An MFL was present in 4/8 cases and was cut before determining the native knee kinematics, to exclude its contribution.

The dimensions of the grid laid over the tibial footprint of the PCL had a mean length of 16.5 ± 2.4 mm, and a mean width of 12,2 ± 1.3 mm. The mean total surface area of the tibial footprint was 178 ± 32 mm^2^.

### Contribution of the total PCL to the restraint of posterior tibial translation in different flexion angles

**Figure 2:**
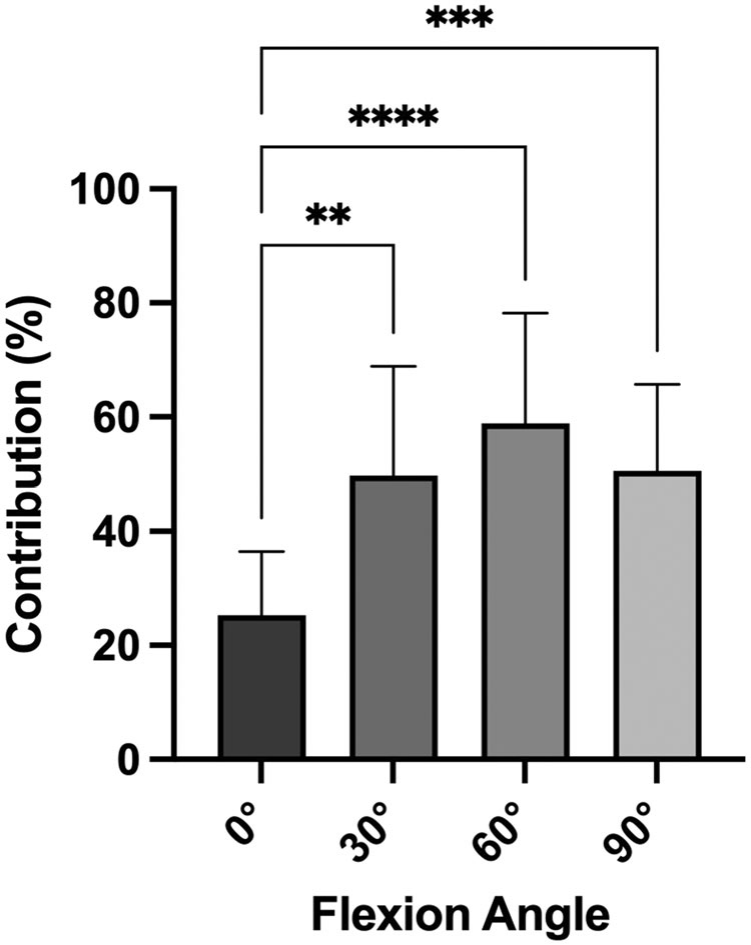
Relative contribution of the total posterior cruciate ligament to restricting posterior tibial translation in different flexion angles. **p < 0.01; ***p < 0.001; ****p < 0.0001.

The complete PCL was found to contribute 25.3 ± 11.1% in 0° of flexion, 49.7 ± 19.2% in 30° of flexion, 58.9 ± 19.3% in 60° of flexion and 50.6 ± 15.1% in 90° of flexion, to the restriction of a posterior drawer force. The contribution of the PCL to restricting posterior tibial translation was found to be significantly (*p *≤ 0.05) lower in 0° of flexion (Figure 2), compared to 30° (24.4%; 95% CI 9.8–39.0), 60° (33.6%; 95% CI 19.0–48.2) and 90° (25.3%; 95% CI 10.7–39.9).

### Sequential cutting of the tibial footprint of the PCL

**Figure 3:**
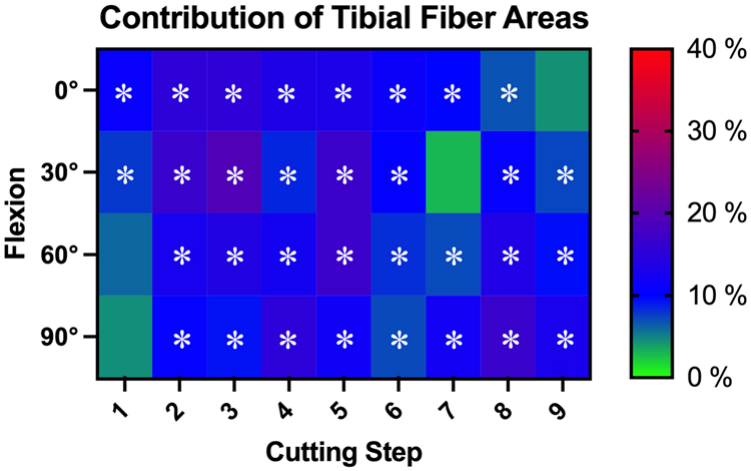
Heatmap visualization of the contribution of each cutting step of the tibial footprint of the posterior cruciate ligament (PCL) in restricting posterior tibial translation in different flexion angles. The contribution of each cutting step is presented as the mean percentage of the contribution of the total PCL. Significant contributors (*p* < 0.05), as determined by statistical analysis, are marked by an asterisk.

Depending on the flexion angle, every cut area of the tibial PCL footprint was shown to be a significant restrictor of posterior tibial translation (*p *< 0.05; Figure 3). Significant contributions of the fibre areas ranged from 6.8 ± 5.8% (area 8) to 15.4 ± 11.5% (area 3) in 0° of flexion. In 30° of flexion, significant contributions of the cut fibre areas ranged from 7.3 ± 7.6% (area 9) to 19.2 ± 17.3% (area 3). In 60° of flexion, significant contributions of the cut fibre areas ranged from 7.2 ± 5.7% (area 7) to 17.0 ± 8.2% (area 5). In 90° of flexion, significant contributions of the cut fibre areas ranged from 7.2 ± 5.9% (area 6) to 16.6 ± 12.5% (area 5).

### Analysis of fibre area contribution regarding row and column

**Figure 4:**
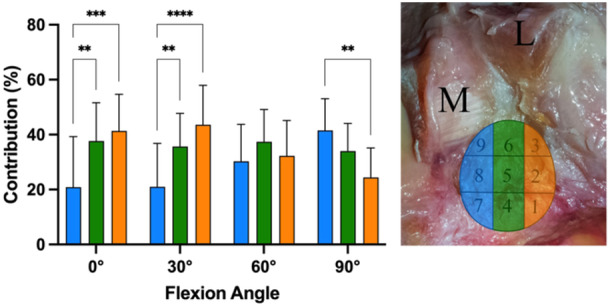
Fibre contributions grouped into columns from medial (M) to lateral (L). **p < 0.01; ***p < 0.001; ****p < 0.0001.

When investigating the fibre contributions in columns from medial to lateral (Figure 4), in 0° of flexion, the most medial areas (7/8/9, mean contribution 20.9 ± 18.4%) showed significant (*p* < 0.05) lower contribution in the restraining of a posterior tibial translation force in comparison to the central fibre areas (areas 4/5/6; MD 16.8%; 95% CI 6.8–26.7) and lateral fibre areas (areas 1/2/3; MD 20.4%; 95% CI 10.5–30.4). Similarly, in 30° of flexion, the most medial areas (mean contribution 21.0 ± 15.8%) showed significantly (*p* < 0.05) lower contribution to restriction of a posterior tibial translation force in comparison to the central fibre areas (MD 14.7%; 95% CI 4.8–24.6) and lateral fibre areas (MD 22.6%; 95% CI 12.7–32.6) as well. Conversely, in 90° of flexion, the most medial areas (mean contribution 41.5 ± 11.6%) showed significantly (*p* < 0.05) higher contribution in comparison to the lateral areas (MD 17.1; 95% CI 7.2–27.1). The highest contributions were found for the lateral fibre areas at 0° (41.4 ± 13.3%) and 30° (43.6 ± 14.3%), for the central areas at 60° (37.4 ± 11.7%) and the medial areas at 90° (41 ± 11.6%).

**Figure 5:**
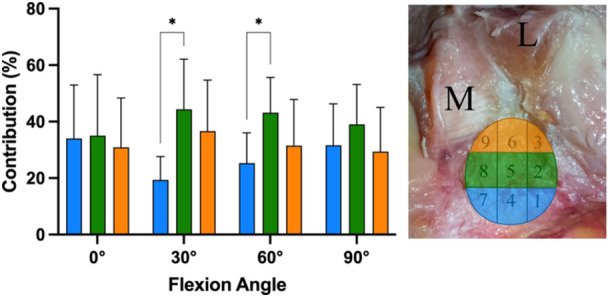
Fibre contributions grouped into rows from posterior (blue shade) to anterior (orange shape). *p < 0.05; M, medial; L, lateral.

When investigating the fibre contributions in rows from posterior to anterior (Figure 5), the central fibre areas showed significantly (*p* < 0.05) higher contribution in comparison to the posterior fibre areas, in 0° (MD 25.0%; 95% CI 7.4–42.6), and 30° (MD 17.8%; 95% CI 3.9–31.7). No significant differences were found between central and anterior fibre rows. The highest contributions were found for the central fibre areas in all flexion angles (mean contributions of 35.0%–44.3%).

### Effect of PCL cutting on anterior tibial translation force

Cutting of the PCL did not result in a statistically significant drop in anterior tibial translation force, indicating no relevant effects that are random and not caused by the sequential cutting.

### Influence of the cutting sequence

No statistically significant difference was found between a cutting sequence starting either from lateral or medial.

## DISCUSSION

The most important finding of the present study is that all fibre areas in the tibial footprint of the PCL are significant contributors to restraining a posterior tibial translation force. Of these fibre areas, the central areas showed the highest contribution, in comparison to the anterior and posterior fibre regions. Furthermore, the lateral fibre areas presented a higher contribution close to extension (0–30°), while the medial fibre areas showed a higher contribution in knee flexion (90°), indicating a reciprocal tensioning pattern of the PCL.

Multiple anatomical studies investigated the tibial footprint of the PCL, with varying descriptions, possibly due to the subjective nature of anatomy and the use of different methods (e.g., arthroscopic vs. direct visualization), ranging from oval to trapezoid shapes [2, 3, 17, 48]. However, it is generally accepted that the anterolateral bundle (ALB) is situated more anteriorly and laterally, in comparison to the posteromedial bundle (PMB). Some authors, however, argue that the PCL is a continuum of fibres, not distinguishable into bundles [9, 25, 35, 40]. In the present study, the tibial footprint of the PCL was identified as an irregular round to oval shape with a mean length of 16.5 ± 2.4 mm, and a mean width of 12,2 ± 1.3 mm, without a clear delineation into two bundles. The total surface covered by the tibial insertion site of the PCL was 178 ± 32 mm^2^, which is comparable to previously published data [3, 21]. In comparison, the surface of the femoral footprint of the PCL was measured to be 340.3 ± 78.1 mm^2^, being approximately twice as large [30], which might result in a different distribution of the contribution of different fibre areas. Indeed, in a recent biomechanical study, a specific fibre area in the femoral PCL footprint was found to be the main restrain of a posterior tibial force [11]. This fibre area inside the femoral footprint was rectangular in shape, and only a fraction of the total fibre area of the femoral footprint in size. This might explain that although the areas of the femoral and tibial footprint differ in size, the fibre areas contributing mainly to the restriction of PTT might actually be similar in size. Based on the combined results of the present study, and the previous study, a flat reconstruction of the PCL with rectangular femoral tunnel, and round tibial tunnel could be biomechanically advantageous [12].

Previous biomechanical studies investigating the PCL mainly focused on the ALB and PMB during sequential cutting. A robotic biomechanical study on cadaveric knee specimens found no significant differences between the contribution of the ALB and PMB in restricting posterior tibial translation between 0° and 90° [18]. Another robotic study found that isolated sectioning of the ALB and PMB led to significant, but small increases in posterior tibial translation (0.9 ± 0.6 mm, and 2.6 ± 1.8 mm respectively in 90° of flexion) [27]. Only complete sectioning of the PCL led to relevant increases in posterior tibial translation (11.7 ± 4.0 mm in 90° of flexion), which may be explainable by the force‐controlled test protocol, in which only complete cutting of a structure has large effects, while partial cutting only has minor effects [28, 36, 38]. In the present study, which focused exclusively on the tibial footprint, all of the investigated fibre areas were significant contributors to restricting a posterior tibial translation force. However, the lateral (1/2/3) and central fibre areas (4/5/6) showed significantly higher contribution in restraining a posterior tibial translation force, in comparison to the most medial areas (7/8/9), in 0° and 30° of flexion. Conversely, in 90° of flexion, the most medial areas showed significantly higher contribution in comparison to the lateral areas. This finding is in accordance with a previous biomechanical study, which found that a PCLR with a medialized tibial tunnel position is subject to increased graft forces in flexion angles >65%, in comparison to a lateralized tibial tunnel position [37]. In summary, these findings indicate a reciprocal tensioning behaviour of the PCL, dependent on the flexion angle of the knee, as described by multiple previous authors [1, 27, 43].

The present study is of clinical relevance due to its implications for clinical practice. Controversy exists regarding the most optimal tibial graft placement for PCLR [53, 56]. Typically, transtibial drilling is performed, placing a K‐wire centrally into the tibial footprint, under fluoroscopic or arthroscopic control [7, 20, 31]. Alternatively, in their landmark paper, Clancy et al. advocated for placement of the K‐wire slightly inferolateral to the femoral footprint, so that when tension is applied, the graft is pressed to the superomedial aspect of the tunnel, coinciding with the centre of the tibial footprint [8]. Furthermore, a tibial inlay technique or double‐bundle technique can be performed [4, 29, 51]. Although initial favourable biomechanical results of these techniques were reported [5], clear clinical superiority of either technique could not be established [32, 53, 55]. Based on the findings of the present study, tibial tunnel placement of the PCL graft should result in the final graft being located centrally in the anatomical tibial footprint, regardless of the applied technique. A malposition of the tibial tunnel to either the medial or lateral side might lead to over‐constraint, either in flexion or extension.

Several limitations have to be considered when interpreting the results of the present study. As with most cadaveric biomechanical studies, knee specimens of older age were used for testing. Small areas were dissected during sequential cutting of the tibial footprint, which makes it possible that cuts were performed either larger or smaller than intended, which might explain some variation in the data. Additionally, by cutting different areas in the same structure, the first principle of superposition has been violated [46, 57]. To compensate for this limitation, the cutting sequence was randomized. Additionally, in studies investigating different fibre areas of a single ligament, superposition was used as well [24, 26, 33]. In the present study, only position‐controlled testing was performed. How cutting of the individual areas would translate to increased posterior tibial translation (in mm) was not assessed. However, it was shown in previous studies, that in force‐controlled studies, the instability produced by a cutting step is dependent on the previous cut, and the last structure to be cut typically leads to a major increase in laxity [28]. By using the principle of superposition, the contribution of each cut structure in restricting PTT, irrespective of the cutting order, could be determined in the present study [46, 57]. However, a future study could investigate the areas of this study with a force‐controlled test protocol to verify the findings. Finally, only posterior tibial translation was examined in this study. Although the PCL was shown to additionally restrict internal/external rotation, this effect is minimal and of questionable clinical relevance [27]. Therefore, the present study refrained from investigating the contribution of the different fibre areas on tibial rotation, to minimize the duration of the test protocol and thereby unnecessary loading of the specimens.

## CONCLUSION

The central row areas in the tibial footprint of the PCL were identified to be the main contributors inside the tibial footprint, while, depending on the flexion angle, the medial or lateral column fibre areas showed a higher contribution. These findings might inform the clinician to place a PCL graft centrally into the tibial footprint during reconstruction.

## SOCIAL MEDIA SUMMARY

Fibres in the tibial footprint of the posterior cruciate ligament were found to contribute differently, depending on flexion angle, in restricting posterior tibial translation, in this robotic biomechanical investigation performed at @UK_Muenster.

## AUTHOR CONTRIBUTIONS

*Conception and design*: Adrian Deichsel and Christoph Kittl. *Testing and data acquisition*: Adrian Deichsel, Wenke Liu and Alina Albert. *Statistical analysis*: Adrian Deichsel and Thorben Briese. *Writing*: Adrian Deichsel. *Proofreading*: Adrian Deichsel, Thorben Briese, Michael J. Raschke, Christian Peez, Elmar Herbst and Christoph Kittl. *Internal review*: Thorben Briese, Michael J. Raschke, Christian Peez and Elmar Herbst. *Supervision*: Michael J. Raschke.

## CONFLICT OF INTEREST STATEMENT

Elmar Herbst is Deputy Editor‐in‐Chief for the Knee Surgery, Sports Traumatology and Arthroscopy (KSSTA). Adrian Deichsel is the Web Editor for the Knee Surgery, Sports Traumatology and Arthroscopy (KSSTA). The remaining authors declare no conflicts of interest.

## ETHICS STATEMENT

The specimens were dissected and biomechanically tested under the approval of the Institutional Ethics Committee of the University of Muenster (File number 2023‐407‐f‐S).

## Data Availability

Data are available from the corresponding author upon reasonable request.
